# Multi-Receptive Field Soft Attention Part Learning for Vehicle Re-Identification

**DOI:** 10.3390/e25040594

**Published:** 2023-03-31

**Authors:** Xiyu Pang, Yilong Yin, Yanli Zheng

**Affiliations:** 1 School of Software, Shandong University, No. 1500, Shunhua Road, High-Tech Industrial Development Zone, Jinan 250101, China; 2 School of Information Science and Electrical Engineering, Shandong Jiaotong University, No. 5001, Haitang Road, Changqing District, Jinan 250357, China

**Keywords:** vehicle re-identification, multi-receptive field, part-level features

## Abstract

Vehicle re-identification across multiple cameras is one of the main problems of intelligent transportation systems (ITSs). Since the differences in the appearance between different vehicles of the same model are small and the appearance of the same vehicle changes drastically from different viewpoints, vehicle re-identification is a challenging task. In this paper, we propose a model called multi-receptive field soft attention part learning (MRF-SAPL). The MRF-SAPL model learns semantically diverse vehicle part-level features under different receptive fields through multiple local branches, alleviating the problem of small differences in vehicle appearance. To align vehicle parts from different images, this study uses soft attention to adaptively locate the positions of the parts on the final feature map generated by a local branch and maintain the continuity of the internal semantics of the parts. In addition, to obtain parts with different semantic patterns, we propose a new loss function that punishes overlapping regions, forcing the positions of different parts on the same feature map to not overlap each other as much as possible. Extensive ablation experiments demonstrate the effectiveness of our part-level feature learning method MRF-SAPL, and our model achieves state-of-the-art performance on two benchmark datasets.

## 1. Introduction

The vehicle re-identification (Re-ID) task identifies the same vehicle from multiple nonoverlapping cameras in surveillance systems. This task is particularly useful when a car’s license plate is occluded or cannot be seen clearly. In these scenarios, the vehicle Re-ID method can effectively locate the vehicle of interest from the monitoring database, which has important applications in intelligent transportation, public safety, smart cities, and other fields. In recent years, vehicle Re-ID has received increasing attention from the computer vision community.

**Figure 1:**
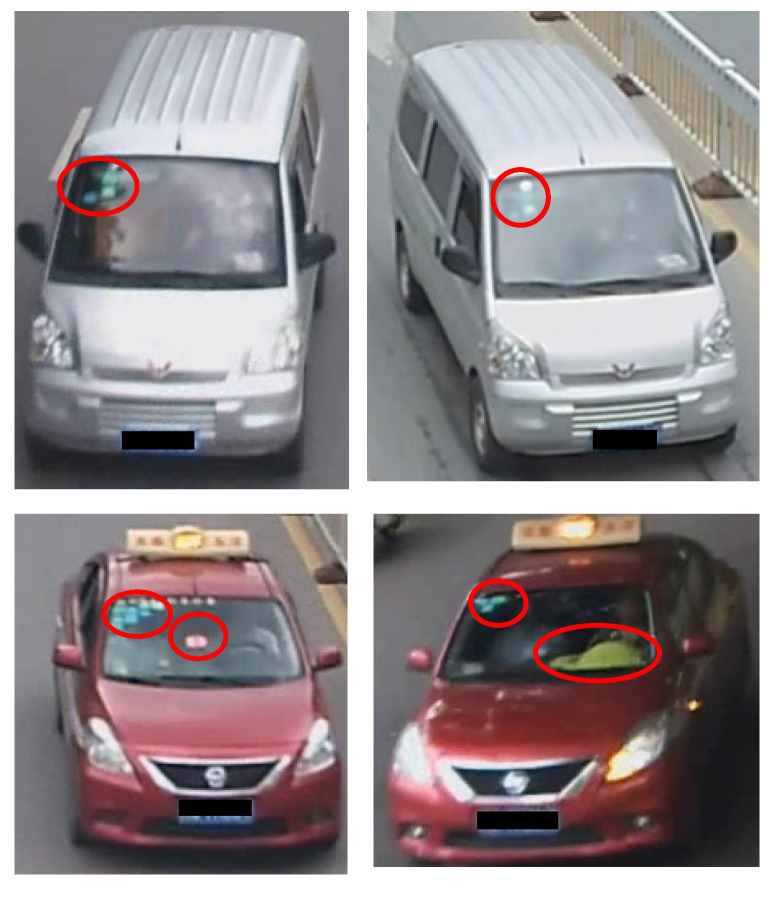
The four cars are divided into two groups: the silver-white cars at the top and the red cabs at the bottom. Each group is comprised by different vehicles of the same model. The detailed information distinguishing each vehicle is marked with red circles; for example, the car in the lower left has rows of stickers including annual inspection signs and a small red screen, while the car in the lower right has three stickers and a yellow item. These details can completely distinguish the two red cars.

Due to drastic changes in illumination, occlusion, resolution, viewing angle, and background, vehicle Re-ID is still a very challenging task, particularly when the vehicle images are obtained from a large number of different cameras. To address this Re-ID task, many deep learning models [1,2,3] for extracting global vehicle information have been proposed in recent years. Although these works have achieved remarkable success in vehicle Re-ID tasks, since global feature learning only captures the most important information representing different identities, the identification ability of global features tends to decline severely when the differences in vehicle appearance are not significant. As shown in Figure 1, different vehicles belonging to the same model may look quite similar. We can distinguish these challenging vehicle image samples by subtle clues, such as the annual inspection signs and decorations marked by the circle shown in Figure 1. Therefore, learning rich fine-grained local features is crucial for vehicle Re-ID tasks.

**Figure 2:**
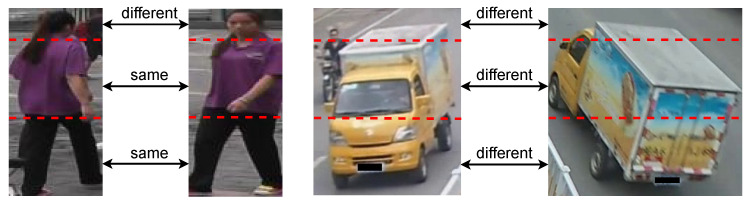
The two images on the left are of the same pedestrian taken from different viewpoints. It can be intuitively seen that the color of the clothes worn by the pedestrian does not change drastically from different viewpoints, and when the pedestrian images are divided vertically into three parts, there are still many commonalities between the corresponding parts of the two images. The two images on the right are of the same vehicle taken from different viewpoints, and it can be seen that the change in viewpoint causes drastic changes in the appearance of the same vehicle, and that the misalignment between the corresponding parts is more severe.

Recently, part-based models [4,5,6,7] have made great progress in learning effective local feature representations for pedestrian Re-ID and have obtained highly promising results. By horizontally dividing one feature map into multiple parts in space, these models can mine fine-grained discriminative features on each part. Aggregating all part-level features can effectively identify pedestrians. Since person and vehicle Re-ID tasks are conceptually similar and both of them belong to the image retrieval problem, techniques from one task can usually be adapted to the other. To solve the problem of high similarity in Figure 1, Ref. [8] divided the feature maps of vehicle images along various directions to extract rich fine-grained local features. They applied the most advanced methods of pedestrian Re-ID to vehicle Re-ID. However, on the one hand, the change in vehicle appearance from different perspectives is much larger than that of pedestrians. As shown in Figure 2, the texture or color of the clothes worn by a person does not change drastically under different viewing angles, meaning that the images of the same person from different cameras will always have more in common and can be roughly spatially aligned, so that the body can be vertically segmented into several parts to extract part-level features. By contrast, the appearance of the same vehicle can change drastically due to the change in viewpoint, and the misalignment of parts is more severe than that of pedestrians, such that a simple rigid spatial division cannot align vehicle parts well enough to learn the part-level features effectively. On the other hand, the simple rigid division of feature maps breaks the semantic continuity within parts.

To overcome the above-described challenges, some methods [9,10,11,12] focus on enabling the networks to identify the vehicle perspective and learn the fine-grained information related to the perspective through vehicle key point detection, parsing networks, and pose estimation. These methods solve the above problems to some extent but increase complexity and rely on additional annotations. In addition, other methods use attention mechanisms to effectively mine identity-related salient information. Ref. [13] enhanced the discriminative power of the features on two branches by using nonlocal spatial attention and channel attention. Although these methods can effectively discover salient information globally, they cannot find rich detailed clues. Analytically, we find that an effective part-level feature learning mechanism for vehicle Re-ID should follow three criteria: (1) the detected parts/regions should be aligned and maintain internal consistency; (2) the detected parts/regions should be semantically diverse to cover as much discriminant information as possible in vehicle images; (3) The detected part semantics should be multilevel because receptive fields of different sizes can capture part information with different semantic levels. To meet these demands, we propose a model called multi-receptive field soft attention part learning (MRF-SAPL) for part-level feature learning. without the need for additional annotations, MRF-SAPL locates parts under multiple receptive fields and learns rich, multi-semantic-level part-level features associated with vehicle identities.

In the MRF-SAPL model, the backbone network is extended to a series of ordered branches, one of which is a global branch for learning global features, and the rest are local branches for learning part-level features. Each local branch mines multiple part-level features with a specific semantic level under a receptive field, so that multiple local branches can obtain enough part-level features with different semantic levels from the entirety of the vehicle image. within each local branch, we use the soft attention part learning (SAPL) module to learn to locate part positions and extract part features. Specifically, first, the final feature map output by a local branch is adaptively divided into several internal semantically continuous parts/regions using soft attention. The adaptive division of regions can automatically align the corresponding vehicle parts from different images. Second, to ensure that the multiple parts extracted by the same branch are semantically irrelevant, we propose a new loss function called the overlapping region penalty (ORP) to force the corresponding regions of different parts on the feature map to not overlap each other as much as possible in order to obtain parts with different semantic patterns. Finally, after positioning the regions where the parts are located, we use a part feature extractor to extract the corresponding part features from each part region. Our contributions can be summarized as follows:

(1) We propose a multi-receptive soft attention part learning (MRF-SAPL) model for vehicle Re-ID that does not require rigid space partitioning or additional labeling and can flexibly discover enough part-level features with multiple semantic levels;
(2) To align the vehicle part features from different images, we exploit soft attention to adaptively divide the space of the feature map to obtain the locations of parts with internal semantic continuity;
(3) Extensive experimental results show that a higher performance can be obtained compared to that of other state-of-the-art methods on two large datasets, where a new loss function, ORP, is proposed to force each local branch of MRF-SAPL to semantically learn complementary part-level features.

## 2. Related Work

### 2.1. Local-Based Re-ID

The design of existing Re-ID methods is mainly based on handcrafted features [14,15], metric learning [16,17,18] and deep learning networks [5,9,19,20,21,22,23,24,25,26,27]. Some recent approaches learn features at the part level and achieve state-of-the-art performance in Re-ID tasks. Existing part-based Re-ID methods can be generalized into two categories: methods with external cues and partition-based methods.

Refs. [10,12,28] used external cues utilize human parsing, pose estimation, and object segmentation to precisely align body parts under the supervision of additional semantic labels. Miao et al. [12] learned the visibility of body parts using pose landmarks and extracted useful features for pedestrians using the generated attention masks. Gao et al. [10] learned part features with the help of attention maps guided by pose estimation and trained the visibility of parts through pseudolabels generated by graph matching. He et al. [28] introduced an object detection network to generate the ROI (region of interest) for each vehicle part and then projected the ROIs into the global feature map generated by a global module to capture local information. However, the required external cues limit the usage and robustness of their method in practical deployments. By contrast, our model can align the corresponding parts of different vehicles using only identity labels under the supervision of the overlapping region penalty (ORP) constraint.

Common segmentation-based models mainly align body parts by rigidly segmenting images/feature maps. Sun et al. [5] horizontally partitioned the final feature map output by the network to learn fine-grained part-level features of pedestrians from each region. Chen et al. [8] divided the feature maps of vehicle images in various directions to fully mine fine-grained local features. Although these methods match part features by region partitioning without using external labels and models, they assume that the same part appears at the same location in different images, making it difficult to overcome the serious spatial misalignment problem inherent in vehicle Re-ID. Recently, Li et al. [29] adaptively learned discriminative body part features for occluded person re-identification tasks by enhancing interpart associations from a global perspective through a transformer encoder-decoder architecture. Both our method and the method of Ref. [29] can adaptively align parts to suppress the spatial misalignments. Different from Ref. [29], MRF-SAPL can generate aligned parts without relying on the complex transformer architecture.

### 2.2. Multiscale Features

Convolutional neural networks extract the features of the target in a layer-by-layer abstract manner through the convolution layer and the pooling layer. The design of the receptive field size has an important impact on the performance of the networks. Small receptive fields can only observe local information; in contrast, large receptive fields can only observe global information. Therefore, researchers have designed various multiscale model architectures to capture features at different semantic levels. He et al. [30] proposed a spatial pyramid pooling network that can obtain fixed-size feature maps and capture information at different scales through different downsampling steps. Zhao et al. [31] proposed the pyramid scene parsing network (PSP Net) that utilizes downsampling and upsampling operations to extract local and global information, making scene recognition more reliable. The Inception module proposed by Szegedy et al. [32] consists of four parallel channels, namely, 1 × 1 convolution, 3 × 3 convolution, 5 × 5 convolution, and 3 × 3 maximum pooling, which are combined to extract the features of the previous layer of different scales. Tolstikhin et al. [33] proposed a multilayer perceptron Mixer (MLP-Mixer) architecture for computer vision that uses a depthwise separable filter with a maximum receptive field and interchannel parameter sharing to mix tokens to capture global information. Li et al. [34] facilitated visual representation learning via 3 × 3 convolutional static context and contextual self-attention-based dynamic context. In this paper, we let each local branch focus on capturing discriminative information under a specific receptive field through a downsampling operation.

## 3. Method

### 3.1. Network Structure

**Figure 3:**
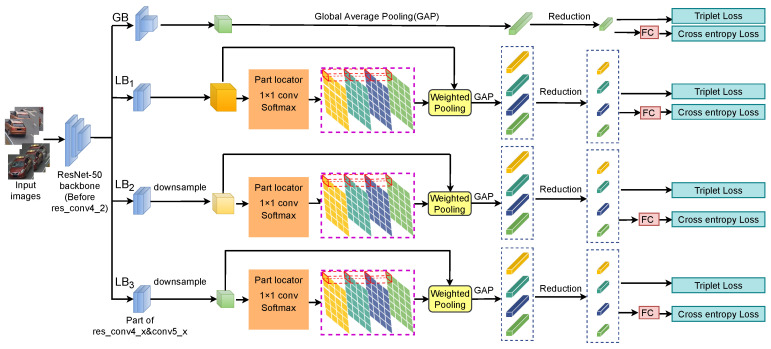
Network architecture of the MRF-SAPL model. It consists of a ResNet-50-based backbone, a global branch for extracting global information, and three local branches ($LB_{1}$, $LB_{2}$, $LB_{3}$). The three local branches extract part features with different semantic levels under different perceptual fields by the soft attention part learning (SAPL) module that consists of a part locator, an overlapping region penalty (ORP) constraint, and a part feature extractor.

Figure 3 shows the overall network architecture of the MRF-SAPL model, which includes a ResNet-50-based backbone, a global branch for extracting global information, and three local branches ($LB_{1}$, $LB_{2}$, $LB_{3}$) for extracting part-level information. For the backbone network, we use ResNet-50 [35] as the basis for the construction of feature map extraction. As with previous works [13,36], we further remove the original fully connected layer for multi-loss training and replicate the res_conv4_2 and subsequent blocks to build four independent branches. One branch is the global branch, and the others are local branches.

The global branch learns a compact global feature representation. In this branch, we use the downsampling convolution layer with a step size of 2 in the res_conv5_1 block and conduct the global average pool (GAP) [37] operation on the final output feature map to obtain a 2048-dimensional feature vector. The dimension of the vector is further reduced to 256 through a dimensionality reduction module that consists of a 1 × 1 convolution layer, a batch normalization layer and a ReLU layer. We use the subnetwork composed of the backbone network and the global branch as the baseline network (baseline) in our experiments.

Intuitively, with the change in the receptive field size, human beings naturally observe an object from different semantic levels. Integrating discriminative information at different semantic levels can help people better identify objects. Therefore, in our network, we introduce three local branches to capture the semantics at different levels to obtain a large amount of discriminative information related to vehicle identities. To preserve enough detailed information, in all local branches, we do not use the downsampling operation in the res_conv5_1 block to provide appropriate space for the change in the receptive field. For each local branch, we first change the resolution of the final feature map to obtain the feature map under a specific receptive field. On the obtained feature map, a part locator uses soft attention to locate the internal semantic continuous parts and uses the ORP constraint to make the semantic patterns between parts different. Then, a part feature extractor generates the corresponding part features according to the positions of the parts on the feature map. Finally, we use the GAP operation on a part feature to obtain a 2048-dimensional feature vector, and the dimension of the vector is further reduced to 256 by a similar dimension reduction module in the global branch for cross entropy loss and triple loss.

The 256-dimensional feature vectors from the global and the three local branches are combined as the final feature representation for the vehicle Re-ID task. The global branch learns the overall discriminative information of vehicles, and the local branches learn the local information at different semantic levels. The global and local branches complement and cooperate with each other to improve their performance. Combining global features with local features can construct a more robust feature representation.

### 3.2. Soft Attention Part Learning Module

Some methods [10,12,38,39,40] train detection models with part labels to detect part locations and extract part-level features. However, it is difficult to collect the additional labels required by these methods. Our proposed SAPL module does not require any labels related to parts and can adaptively learn the locations of the parts on the feature map and extract the part features. It consists of a part locator, an overlapping region penalty constraint and a part feature extractor. After the final feature map of a local branch passes through the SAPL module, we obtain the position of each part on the feature map and a constant number of part-level features.

We define the feature map generated by an image via a backbone and a local branch as a three-dimensional tensor *T* with the size of h×w×c (*h*, *w*, and *c* represent the channel height, width, and channel number, respectively). We define the activation vector viewed along the channel dimension as pixel *z*, which indicates the semantic information of its location. The purpose of the part locator is to locate the spatial positions of the parts on the *T* and to ensure the continuity and consistency of the internal semantics of the parts. Therefore, according to the semantic similarity between pixels, we use soft attention to assign them to each part. Specifically, the part locator is implemented by a fully connected layer followed by a softmax function, which is given by:

(1) $$P\left(P_{i}|z\right)=\mathrm{softmax}\left(W^{T}z\right)=\frac{\exp\left(W_{i}^{T}z\right)}{\sum_{j=1}^{p}\exp\left(W_{j}^{T}z\right)},$$

where $P(P_{i}|z)$ is the prediction probability of part $P_{i}$ at the *z* of the feature map *T* and *W* is the weight matrix of the fully connected layer. *p* is the number of vehicle parts.

After applying the part locator on each pixel of *T*, we obtain a set of attention maps $A=\left\{A_{i}|i=1,\ldots ,p\right\}$, where $A_{i}\in R^{h\times w}$ indicates the position of the i-th part on the feature map *T* and can be reshaped into a vector with dimension hw. To obtain multiple parts with different semantic patterns in a branch, rather than just focusing on the main discriminant area, the corresponding positions of different semantic parts should have a small overlap in space. Therefore, the overlapping region penalty (ORP) constraint is proposed to measure the area of the overlapping region of *A* that is defined as:

(2) $$L_{\mathrm{orp}}=\sum_{i\neq j}\frac{A_{i}^{T}A_{j}}{{∥A_{i}∥}_{2}\cdot {∥A_{j}∥}_{2}},$$

where ${∥\cdot ∥}_{2}$ is the L2 norm. The ORP constraint adaptively softly divides the semantic space and generates multiple parts with different semantics. The combination of the part locator and ORP constraint has two beneficial effects for part segmentation. On the one hand, semantically similar features from a particular part are encouraged to be grouped together so that a strong part locator can be learned and corresponding parts from different images can be aligned. On the other hand, different semantic patterns between parts are encouraged to obtain multiple semantic complementary parts.

After obtaining the attention map of each part on the feature map, the part feature extractor generates the corresponding features for each part. Given that pixel *z* on the feature map belongs to the prediction probability of part $P_{i}$, the part feature extractor generates feature $f_{i}$ of the part by weighted pooling that is calculated using the following formula:

(3) $$f_{i}=\frac{\sum_{z\in T}P\left(P_{i}|z\right)\times z}{C_{i}+\epsilon },$$

where divisor $C_{i}$ is the accumulation of $P(P_{i}|z)$ on *T* and represents the salience of each part on the image. It should be noted that if a vehicle part is not visible in an image, all values of the attention map generated by the part locator for the part are close to 0. Hence, to avoid using 0 as a divisor when $C_{i}$ is 0, ϵ is a small constant, which is set to 0.05 in our implementation.

### 3.3. Multi-Receptive-Field Granularity

Humans can capture different levels of semantics of a vehicle (such as vehicle type, lamp shape, and annual inspection sign) under different receptive fields (such as viewing distance or image resolution). Some types of semantics (e.g., with or without annual inspection) may be easier to capture in small receptive fields, while others (e.g., car door style) may be easier to capture in large receptive fields. Inspired by this, we propose a multi-receptive field soft attention part learning (MRF-SAPL) model to capture discriminative information on different semantic levels.

In MRF-SAPL, each local branch corresponds to a receptive field granularity, and we distinguish different receptive field granularities through different resolutions of the final feature maps of the local branches. According to the previous description, when a vehicle image passes through all local branches, we obtain a set of feature maps $T_{all}=\left\{T_{m}|i=1,2,3\right\}$, where $T_{i}\in R^{h\times w\times c}$ includes h×w pixels (*h*, *w*, *c* represent the height, width, and number of channels, respectively). For the $m_{th}$ granularity, we perform spatial average pooling with a downsampling factor m on the $m_{th}$ feature map of $T_{all}$ and obtain the downsampled feature map $T_{m}^{{}^{\prime }}\in R^{h_{m}\times w_{m}\times c}$ of $h_{m}\times w_{m}$ pixels, where $h_{m}=h-4\left(m-1\right)$ and $w_{m}=w-4\left(m-1\right)$. The factorized feature map set is $T_{all}^{{}^{\prime }}=\left\{T_{m}^{{}^{\prime }}|i=1,2,3\right\}$. We apply the SAPL module separately on all feature maps of $T_{all}^{{}^{\prime }}$ to obtain multiple part features on different semantic levels.

### 3.4. Multitask Training

Multitask learning combines several related subtasks for overall learning and has been shown to be effective in Re-ID problems. We train our network by three types of supervision, i.e., the cross-entropy loss, the triplet loss, and the ORP loss $L_{orp}$ in Equation (2). The cross-entropy loss is expressed as:

(4) $$L_{id}=-I_{k=y}\log\left(h\right),$$

where $I_{k=y}$ returns 1 only when the predicted class *k* of a sample is equal to its supervised class *y*; otherwise, it returns 0. *h* is the probability that the sample is predicted to be class *k*.

The triplet loss separates the distance between examples of the same vehicle and the distance between examples of different vehicles by a certain threshold. We adopt the triplet loss with hard mining of Ref. [36]. During model training, *P* vehicles and *K* images of each vehicle are randomly sampled for each mini-batch to meet the triplet loss requirement. The triplet loss can be defined as:

(5) $$\begin{array}{cc} L_{\mathrm{tp}}= & \sum_{i=1}^{P}\sum_{a=1}^{K}\left[\alpha +\max_{p=1,\ldots ,K}{∥a_{i}-p_{i}∥}_{2}\right.\\ \; & {\left.-\min_{n=1,\ldots ,K,j=1,\ldots ,P,j\neq i}{∥a_{i}-n_{j}∥}_{2}\right]}_{+}, \end{array}$$

where α is the margin hyperparameter that controls the differences of intra and inter distances, and $a_{i}$, $p_{i}$, and $n_{j}$ are the feature representations extracted from anchor, positive, and negative samples, receptively.

The cross-entropy loss and the triplet loss are used to supervise the network to learn identity-related global and local features. The overall training loss is formulated by:

(6) $$L=L_{orp}+L_{id}+L_{tp},$$

where $L_{orp}$ prefers that the activated regions of different parts are nonoverlapping, and $L_{id}$ and $L_{tp}$ guide the model MRF-SAPL to activate the image discriminative regions rather than the background.

## 4. Experiments

### 4.1. Datasets and Evaluation Metric

We evaluate our proposed model on the VeRi-776 and VehicleID datasets, which are two mainstream datasets used in vehicle re-identification tasks.

VeRi-776 is the benchmark dataset of the vehicle Re-ID task. It consists of 49,357 images of 776 different vehicles captured by 20 nonoverlapping cameras in various directions and lighting conditions. The training and test sets contain 37,781 images of 576 vehicles and 11,579 images of 200 vehicles, respectively. According to the evaluation protocol in Ref. [2], we employ an image-to-trajectory cross-camera search, that is, using a vehicle image of a camera to search the trajectory of the same vehicle in other cameras. We measure the performance of our proposed model using mean average precision (mAP) and the Top-1 and Top-5 accuracies of cumulative matching curves (CMC).

VehicleID is another data-heavy benchmark consisting of 221,567 images from 26,328 different vehicles, of which 113,346 images from 13,164 vehicles are used for training and the rest are used for testing. The test set is further divided into three subsets of different sizes (small, medium, and large). In the inference phase, for each subset, one image is randomly selected from the images of each vehicle to form the gallery set, and the other images are used as query images. The average result of 10 repeated random samplings is regarded as the performance of our model on the VehicleID dataset. The evaluation indices of the VehicleID dataset are the Top-1 and Top-5 accuracies of CMC.

### 4.2. Implementation Details

Prior to feeding the vehicle images into the MRF-SAPL model, we resize them to 256 × 256 for more detailed information. The weights of the backbone and branches of MRF-SAPL are initialized with ResNet-50 [35] pretrained on ImageNet. During the training phases, we only randomly flip the input images horizontally for data augmentation. By randomly selecting 16 vehicles with 4 images per vehicle, the batch size is set to 64. We set the margin parameter of the triplet loss to 1.2 in all experiments. We choose stochastic gradient descent (SGD) as the optimizer. The initial learning rate is set to 0.01 and decays to 1 × 10^−3^ after 300 epochs and 1 × 10^−4^ after 400 epochs. The total training process lasted for 500 epochs. During testing, we concatenate all dimensionality-reduced feature vectors as a feature representation for each image in the query and gallery sets. The feature representations extracted from the original and horizontally flipped images are summed and normalized as the final vehicle feature embedding for the input image. Our model is implemented on two NVIDIA RTX 2080Ti GPUs using the PyTorch framework.

### 4.3. Comparison with State-of-the-Art Methods

We compared the proposed model MRF-SAPL in this paper with the current methods on the VeRi-776 and VehicleID datasets with the corresponding evaluation indices.

**VeRi-776:** Table 1 presents the comparison of previous methods and our model on the VeRi-776 dataset. Among these methods, Siamese+Path [1] relies on the temporal and spatial information of the vehicle images in the VeRi-776 dataset. TCPM [25] divides the final feature map from the horizontal and vertical directions and uses an external memory module to store partial features to model the global feature vector. Dual+SA [41] uses self-attention to generate attention maps about the vehicle model and vehicle ID and inputs the attention map to the part localization module to obtain the fine region features of ROIs. Relying only on visual information, our proposed model MRF-SAPL achieves 81.5% mAP, 94.7% Top-1 accuracy, and 98.7% Top-5 accuracy. Our model is superior to these advanced methods in terms of mAP and Top-1 accuracy. A good mAP score shows that MRF-SAPL has a stronger ability to retrieve all corresponding images with the same identity in the gallery set, both for different camera attributes and viewpoint changes.

**VehicleID:** We compared the scores of Top-1 and Top-5 on this dataset because each query vehicle has only one corresponding image in the gallery set. The comparison of the results on the Vehicle-ID dataset is shown in Table 2. VAMI [11] utilizes an adversarial training network and vehicle attributes to infer the features of the input vehicle under different viewpoints. PRN [28] utilizes an object detection network to generate the ROI for each vehicle part and extract part features. LRPT+TSAM+CP [47] lets a parameter generator network capable of generating complex image transform regions and a recognizer compete with each other to enhance images. An examination of the results presented in Table 2 shows that our MRF-SAPL outperforms SOTA TCPM by 2.3%, 0.8%, and 1.7% in Top-1 accuracy on small, medium, and large subsets, respectively. Compared with other models, our MRF-SAPL model achieves the best performance. without resorting to additional labels, object detection, and parsing networks, our proposed model can learn rich fine-grained local features for vehicle Re-ID.

### 4.4. Ablation Study

We conducted extensive experiments on the VeRi-776 dataset and compared the performance of different structures to determine the optimal structure of the proposed model.

**Soft attention part learning module.** In Table 3, “+” indicates the combination of different branches. Baseline+$LB_{1}$, Baseline+$LB_{2}$, and Baseline+$LB_{3}$ outperform Baseline by 6.9%, 6.8%, and 4.7% in mAP, respectively. Baseline+$LB_{1}$ (W/O SAPL) means removing the SAPL module from Baseline+$LB_{1}$ and dividing the final feature map of the $LB_{1}$ branch evenly into four parts vertically. We observe a 1.1% decrease in mAP with Baseline+$LB_{1}$(W/O SAPL) compared to Baseline+$LB_{1}$. This demonstrates the effectiveness of our proposed soft-attention part learning module.

**Multi-receptive field granularity**. Our framework contains three local branches with different receptive field granularities, namely fine-grained, medium-grained, and coarse-grained branches, which are responsible for part segmentation and feature extraction under different receptive fields. We investigate the role of multiple receptive field granularities in MRF-SAPL by progressively combining local branches based on the baseline. From Table 3, we can observe that combining two local branches with different receptive field granularities can further improve the performance, and MRF-SAPL using three receptive fields of different sizes to learn part-level features achieves the best performance; this shows that learning part-level features with different semantic-level preferences using different granularities of receptive fields is effective.

**Global branch.** In Table 3, $LB_{1}$+$LB_{2}$+$LB_{3}$ means that the global branch is removed from MRF-SAPL, and only three local branches are used to train the network. At test time, feature vectors from the three local branches are extracted and concatenated to compute a similarity score. Compared with MRF-SAPL, the accuracy of $LB_{1}$+$LB_{2}$+$LB_{3}$ decreases by 1.3% in mAP. This is because the global branch with a larger receptive field can learn the overall discriminant information of vehicles, complementing the local branches that learn fine-grained local discriminant information.

**Multiple local branches.** In our method, we use three local branches to learn part features with different semantic levels from vehicle images; therefore, we would like to know whether it is possible to learn part features with different semantic levels using a single branch. To verify this hypothesis, we can perform spatial soft segmentation on the final feature map of the same local branch under multiple receptive fields and apply the corresponding constraints of the method proposed in this paper. From Table 3, we can observe that Baseline+Single($LB_{1}$+$LB_{2}$+$LB_{3}$) relying on a single local branch has a 4.4% performance drop in mAP compared to MRF-SAPL. This may because using different receptive field granularities to softly divide the space of the same feature map will have different or even the opposite effects on its res_conv5 layer.

**Influence of the number of parts.** To study the impact of the number of parts on the Re-ID accuracy, we introduce several divisions with different numbers of parts. Specifically, we conduct experiments with the 2, 3, 4, and 5 parts. In each experiment, the feature maps on the three local branches are divided into the same number of regions. The experimental results are summarized in Table 4. with an increasing number of parts, mAP first increases, but does not always increase. When the number of parts is equal to 5, mAP starts to decrease. This is because when the spatial of a feature map is too finely divided, some semantic information that is meaningful for vehicle Re-ID will be decomposed into segments that do not have general discriminative abilities. In our proposed method, the number of parts of the final feature maps of all local branches is set to 4.

**Figure 4:**
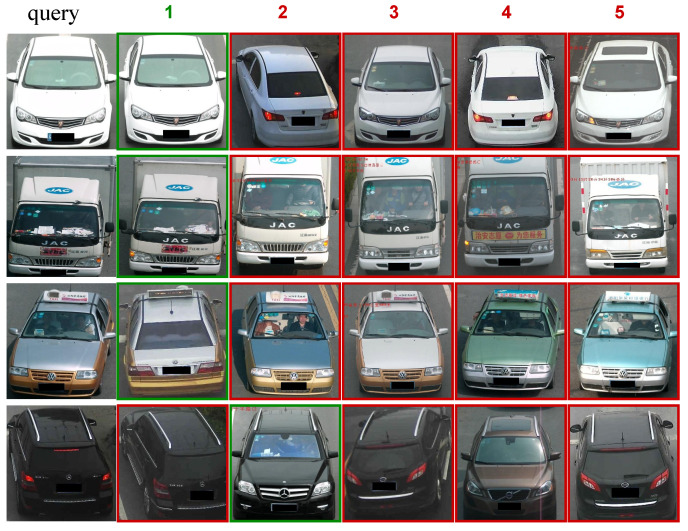
Visualization of the ranking list on the vehicleID dataset. The images in the first column are the vehicle images to query. The remaining images in each row are the Top-5 ranking results retrieved from the gallery that are most similar to the corresponding query image. The retrieved images with the same ID as the query image are shown with the green border, while the error samples are shown with the red border. Note: Some vehicle images in the VehicleID dataset contain Chinese characters for the shooting time and location, such as the characters in the top left corner of the Top-2 image in the second line, and their impact on vehicle recognition can be negligible.

**Figure 5:**
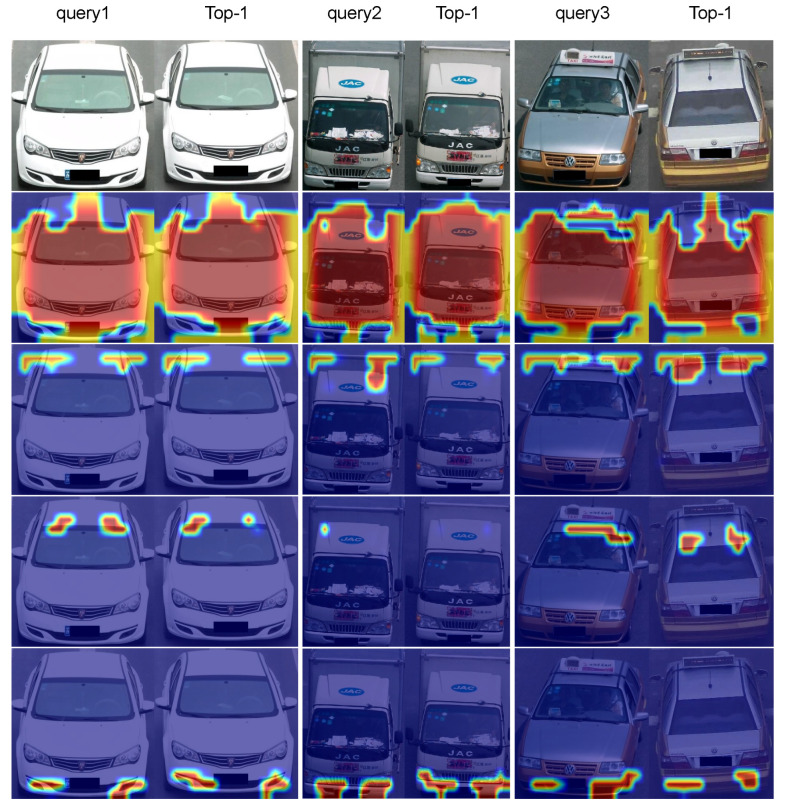
Visualization of attention maps. Each column displays four attention maps generated by the $LB_{1}$ branch of MRF-SAPL for a image. The first and second columns correspond to the attention maps of the query image and the Top-1 image in the first row of Figure 4, respectively. The third and fourth columns correspond to the attention maps of the query image and the Top-1 image in the second row of Figure 4, respectively. The fifth and sixth columns correspond to the attention maps of the query image and the Top-1 image in the third row of Figure 4.

**Vehicle sorting and attention map visualization.** Figure 4 shows the qualitative results of our MRF-SAPL model on the vehicleID dataset, where each query image only has one target image in the gallery set. In Figure 4, the images on the left are the query images, and the images on the right are the Top-5 nearest neighborhoods from the gallery. Figure 5 shows the attention map visualization of the SAPL module in the $LB_{1}$ branch when the number of parts is 4. From Figure 5, we can observe that the four attention maps learned by the SAPL module focus on four different regions: the main area consisting of the lower part of the windshield and the hood, the roof area, the annual inspection mark area in the upper part of the windshield, and the fog lamp area. For the first row in Figure 4, the query image and the Top-3 image are two different vehicles belonging to the same manufacturer and model, with extremely similar appearances. The SAPL module accurately distinguishes them by focusing on the annual inspection mark area. For the third row in Figure 4, Top-4 and Top-5 have large color differences in the main area compared to the query, so they are ranked lower. Although Top-1 and the query are the same vehicle captured under different views, and Top-2 and Top-3 are extremely similar to the query, the SAPL module can distinguish them by focusing on the roof area and the main area, respectively. For the fourth row in Figure 4, the query and Top-1 are two different vehicles belonging to the same manufacturer and model, both of which were captured from a rear view. Top-2 and the query are the same vehicle captured from different perspectives. In this case, the SAPL module has difficulty distinguishing between Top-1 and Top-2 because there is no obvious difference in appearance information between Top-1 and the query. This demonstrates that MRF-SAPL is able to effectively distinguish vehicles with extremely similar appearances in most cases.

## 5. Conclusions

In this paper, we propose a model for part-level feature learning, the Multi-Receptive Field Soft Attention Part Learning (MRF-SAPL) model. The model can learn fine-grained features at multiple semantic levels to effectively distinguish different vehicles with similar appearances. In particular, the soft-attention part learning module (SAPL) in this model does not require any part-related labels and can adaptively learn to localize the locations of the parts on the feature map to suppress severe spatial misalignments in vehicle Re-ID. Furthermore, we obtain parts with different semantic patterns by forcing the regions corresponding to the parts on the final feature map of a local branch to be as nonoverlapping as possible. Our Multi-Receptive Field Soft Attention Part Learning model achieves state-of-the-art performance on two public datasets.

## Figures and Tables

**Table 1:** The mAP, Top-1, and Top-5 on VeRi-776.

Method	mAP	Top-1	Top-5
Siames+Path [1]	0.583	0.835	0.900
VAMI [11]	0.501	0.770	0.908
RAM [42]	0.615	0.886	0.940
EALN [43]	0.574	0.844	0.941
AAVER [44]	0.612	0.890	0.947
PRN [28]	0.743	0.943	0.989
VCAM [40]	0.686	0.944	0.969
SPAN [26]	0.689	0.940	0.976
TCPM [25]	0.746	0.940	0.971
VSCR [45]	0.755	0.941	0.979
LCDNet+BRL [46]	0.760	0.946	0.980
Dual+SA [41]	0.786	0.944	0.992
MRF-SAPL (Ours)	0.815	0.947	0.987

**Table 2:** The Top-1 and Top-5 on Vehicle ID.

Method	Small		Medium		Large	
	Top-1	Top-5	Top-1	Top-5	Top-1	Top-5
DRDL [48]	0.490	0.735	0.428	0.668	0.382	0.616
OIFE [9]	-	-	-	-	0.670	0.829
VAMI [11]	0.631	0.833	0.529	0.751	0.473	0.703
RAM [42]	0.752	0.915	0.723	0.870	0.677	0.845
AAVER [44]	0.747	0.938	0.686	0.900	0.635	0.856
EALN [43]	0.751	0.881	0.718	0.839	0.693	0.814
PRN [28]	0.784	0.923	0.750	0.883	0.742	0.864
SAVER [17]	0.799	0.952	0.776	0.911	0.753	0.883
TCPM [25]	0.820	0.964	0.788	0.943	0.746	0.907
Dual+SA [41]	-	-	-	-	0.738	0.835
SN++ [49]	0.767	0.870	0.748	0.842	0.739	0.836
LRPT + TSAM + CP [47]	0.779	0.935	0.779	0.907	0.745	0.865
MRF-SAPL (Ours)	0.843	0.977	0.796	0.941	0.763	0.916

**Table 3:** Performance comparison of MRF-SAPL with different architecture on VeRi-776.

Method	mAP	Top-1	Top-5
Baseline	0.726	0.918	0.973
Baseline+$LB_{1}$	0.795	0.932	0.985
Baseline+$LB_{1}$(W/O SAPL)	0.784	0.928	0.980
Baseline+$LB_{2}$	0.794	0.938	0.982
Baseline+$LB_{3}$	0.773	0.924	0.983
Baseline+$LB_{1}$+$LB_{2}$	0.813	0.935	0.983
Baseline+$LB_{1}$+$LB_{3}$	0.805	0.935	0.982
Baseline+$LB_{2}$+$LB_{3}$	0.795	0.945	0.982
$LB_{1}$+$LB_{2}$+$LB_{3}$	0.802	0.938	0.982
Baseline+Single($LB_{1}$+$LB_{2}$+$LB_{3}$)	0.771	0.923	0.979
MRF-SAPL (Ours)	0.815	0.947	0.987

**Table 4:** Influence of the number of parts on VeRi-776.

The Number of Parts	mAP	Top-1	Top-5
2	0.801	0.944	0.985
3	0.807	0.939	0.984
4	0.815	0.947	0.987
5	0.802	0.938	0.982

## Data Availability

All data included in this study are available upon request by contact with the corresponding author.
